# A versatile approach to multiple gene RNA interference using microRNA-based short hairpin RNAs

**DOI:** 10.1186/1471-2199-8-98

**Published:** 2007-10-30

**Authors:** Xiaocui Zhu, Leah A Santat, Mi Sook Chang, Jamie Liu, Joelle R Zavzavadjian, Estelle A Wall, Christine Kivork, Melvin I Simon, Iain DC Fraser

**Affiliations:** 1 The Alliance for Cellular Signaling, Division of Biology, California Institute of Technology, Pasadena, CA 91125, USA

## Abstract

**Background:**

Effective and stable knockdown of multiple gene targets by RNA interference is often necessary to overcome isoform redundancy, but it remains a technical challenge when working with intractable cell systems.

**Results:**

We have developed a flexible platform using RNA polymerase II promoter-driven expression of microRNA-like short hairpin RNAs which permits robust depletion of multiple target genes from a single transcript. Recombination-based subcloning permits expression of multi-shRNA transcripts from a comprehensive range of plasmid or viral vectors. Retroviral delivery of transcripts targeting isoforms of cAMP-dependent protein kinase in the RAW264.7 murine macrophage cell line emphasizes the utility of this approach and provides insight to cAMP-dependent transcription.

**Conclusion:**

We demonstrate functional consequences of depleting multiple endogenous target genes using miR-shRNAs, and highlight the versatility of the described vector platform for multiple target gene knockdown in mammalian cells.

## Background

The discovery of RNA interference (RNAi) and its use as an experimental tool has heralded a new era in functional genomics [1,2]. The ability of short interfering RNA (siRNA) to perturb expression of any gene target highlights the enormous potential of this technique. However, siRNA delivery into primary cells, cell lines that are difficult to transfect and delivery to specific cell types *in vivo *remains a key technical issue.

**Figure 1:**
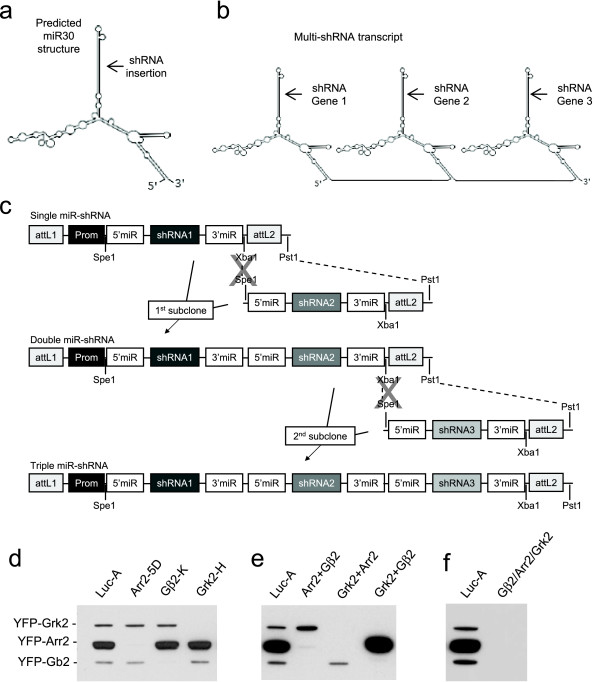
**Expression of concatenated miR30-based shRNAs in a single transcript can promote efficient knockdown of at least three target genes**. (**a**) Predicted secondary structure of the unprocessed miR30 transcript. Arrow depicts the location of the mature miRNA which is replaced with gene specific siRNA sequence in the expressed miR-shRNA. (**b**) Proposed structure of a transcript expressing multiple gene specific miR-shRNAs. (**c**) Subcloning scheme for creation of multi-miR-shRNA transcripts. Prom; any of the pol II promoters listed in Fig. 2a, attL1 + attL2; Gateway recombination sites, 5'miR + 3'miR; flanking sequence derived from human miR30. (**d**) Single CMV promoter-driven miR-shRNAs against arrestin 2 (Arr2), G beta 2 (Gβ2) and G-protein coupled receptor kinase 2 (Grk2) promote potent and specific knockdown when co-expressed with YFP-tagged cDNAs of their respective target genes in HEK293 cells. (**e**) Single transcripts expressing different combinations of double miR-shRNAs promote efficient dual target knockdown. (**f**) A single transcript expressing miR-shRNA against Arr2, Gβ2 and Grk2 retains potency against all three target genes.

A variety of vector-based approaches, which express siRNAs as short hairpin (sh)RNAs, have been developed to permit delivery through viral vectors [3]. Designing such shRNA in the context of a naturally occurring RNA polymerase (pol) II-driven microRNA transcript (miR-shRNA; Fig. 1a) increases the flexibility of this approach allowing for conditional and cell type-specific expression [4-11]. The observation that some endogenous microRNAs (miRNA) are processed from single transcripts containing multiple primary miRNA sequences [12,13] adds to the potential experimental utility of this approach to RNAi. In using RNAi as an experimental tool, the ability to effectively deplete multiple gene targets is vital to address the issue of isoform redundancy, especially in mammalian cell systems. Thus, a flexible vector platform that allows for effective multi-gene knockdown in essentially any cell system would be of great value.

We report the development of a versatile system which permits the knockdown of multiple target genes from a single transcript, and we show efficient depletion of endogenous pairs of signaling genes in the RAW264.7 murine macrophage-like cell line. Importantly, we demonstrate the absolute requirement for multi-gene depletion to observe phenotypes in processes dependent on proteins with redundant isoforms.

## Results and discussion

**Figure 2:**
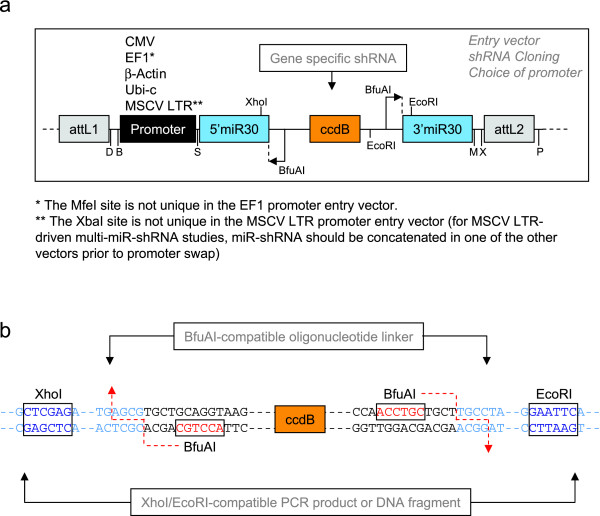
**Entry vector design and miR-shRNA cloning strategy**. **(A) **Schematic showing the architecture of entry vectors for miR-shRNA cloning. Vectors were created with five different promoter options; CMV, EF1, β-actin, Ubiquitin-c and MSCV LTR. Single letters denote the following restriction enzyme sites: D; DpaI, B; BamHI, S; SpeI, M; MfeI, X; XbaI, P; PstI. These sites are unique apart from the exceptions noted below the figure. The ccdB is a counterselection gene toxic to most *E.coli *strains, which reduces parent vector background when cloning shRNAs. **(B) **Sequence details around the shRNA cloning sites demonstrate alternative methods of shRNA insertion into an entry vector. For shRNAs cloned as BfuAI site-compatible linkers (see methods), shRNA sequence is introduced at the junctions of the 5' and 3' miR30 sequence (light blue). For shRNAs subcloned from commercially available whole genome libraries [2, 7], fragments can be subcloned to the XhoI/EcoRI sites (dark blue) within the 5' and 3' miR30 sequence.

To determine if multi-miR-shRNA transcripts could promote efficient and specific knockdown in mammalian cells, we first created plasmids expressing potent miR-shRNAs against the murine orthologs of three genes involved in G-protein signaling: arrestin 2 (Arr2), G-protein coupled receptor kinase 2 (Grk2) and G-protein beta 2 (Gβ2) (see methods and Fig. 2a). Co-transfection of each of these miR-shRNA-expressing plasmids with YFP-tagged cDNAs of all three target genes in HEK293 cells resulted in potent and specific knockdown of the intended target protein (Fig. 1d). Using the premise that multiple miR-shRNAs targeting different genes could be linked in a single transcript (Fig. 1b), we designed our miR-shRNA-expressing plasmids to allow simple concatenation through directional subcloning (Fig. 1c). Using this strategy, we created dual miR-shRNA constructs targeting Arr2+Gβ2, Grk2+Arr2 and Grk2+Gβ2. Co-transfection with YFP-tagged cDNAs of the target genes in HEK293 cells resulted in specific knockdown of each target gene pair, with no apparent loss of shRNA potency (Fig. 1e). Similarly, a construct expressing all three miR-shRNAs led to efficient triple target knockdown (Fig. 1f). To determine if plasmids with multiple miR-shRNAs remain stable in *E.coli*, we performed serial passaging of the triple miR-shRNA construct used in Fig. 1f. Sequencing of clones isolated from up to five rounds of serial passaging found no re-arrangement of miR-shRNA sequences (data not shown). The data in Figure 1 validate the principle of depleting multiple gene targets with a single transcript containing multiple miR-shRNA cassettes, however, such transient expression in a readily transfectable cell line offers little practical utility over the use of multiple siRNAs.

**Figure 3:**
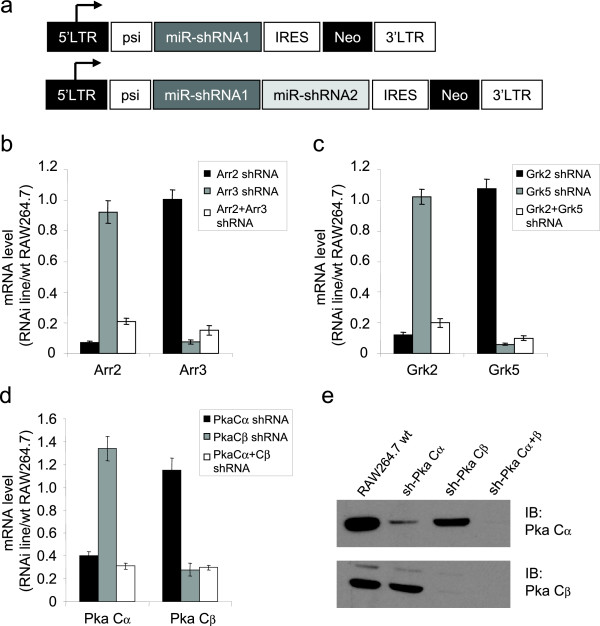
**Stable expression of multi-miR-shRNA transcripts in a murine macrophage cell line promotes efficient knockdown of endogenous target genes**. (**a**) Architecture of retroviral vectors (FBneo) used to stably express single or double miR-shRNAs in RAW264.7 (RAW) macrophages. (**b-d**) Retroviral expression of miR-shRNA can promote efficient and stable knockdown of multiple endogenous target mRNAs; Arrestin 2 and 3 (**b**), Grk2 and 5 (**c**) and Pka Cα and Cβ (**d**) (Data represent averages (± SEM) from at least 2 independent sample sets). (**e**) Western blots from Pka Cα and Cβ RAW cell lines demonstrate >95% knockdown of target proteins.

To determine if this shRNA expression approach could be applied to less tractable cells, we created a comprehensive range of viral vectors to which miR-shRNA cassettes could be transferred by Gateway^® ^recombination (Additional file 1). Using a retroviral vector which bicistronically expresses the miR-shRNA(s) with a neomycin resistance gene (Fig. 3a), we created stable lines of RAW264.7 murine macrophage (RAW) cells expressing single and dual miR-shRNAs against three different pairs of signaling genes; Arr2 and 3, Grk2 and 5 and the cAMP-dependent protein kinase (Pka) catalytic subunits Cα and Cβ. We observed efficient single and dual knockdown of the endogenous target genes in RAW cells using this approach (Fig. 3b–e). These data demonstrate the flexibility of the multi-miR-shRNA approach in effectively targeting three different pairs of signaling genes in a cell line which is intractable to efficient siRNA transfection. Moreover, the ability to deplete multiple genes with redundant functions is an important technical advance in the effort to dissect signaling pathways in cells such as RAW macrophages [14].

**Figure 4:**
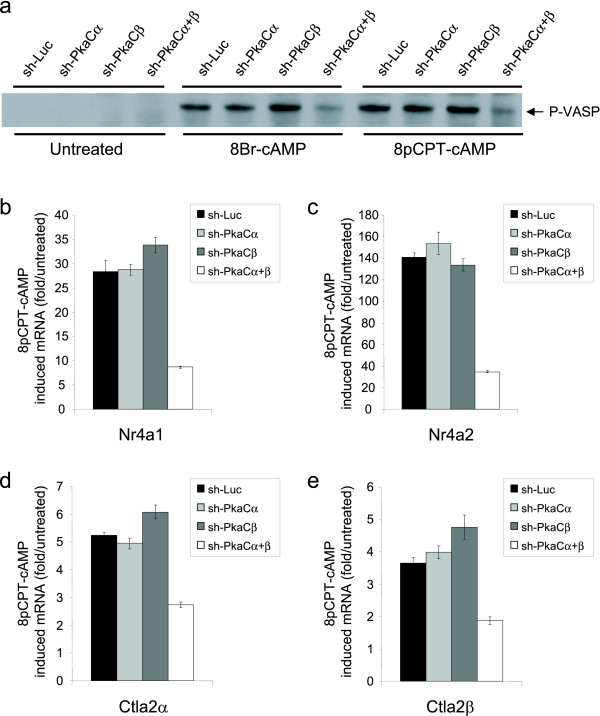
**Efficient knockdown of both PKA alpha and beta catalytic subunits is necessary to attenuate PKA-dependent phosphorylation and transcription**. (**a**) Phosphorylation of the cytoskeletal protein VASP in response to cAMP analogs; combined knockdown of Pka Cα and Cβ is required to attenuate phosphorylation in response to either 8Br-cAMP or 8pCPT-cAMP. (**b-e**) Induction of PKA-dependent transcripts Nr4a1, Nr4a2, Ctla2α and Ctla2β in response to 8pCPT-cAMP. Induction of all 4 transcripts is significantly attenuated only upon depletion of both Pka Cα and Cβ (Data represent averages (± SEM) from 2 independent experiments).

Mammalian cells express two Pka catalytic subunits, Cα and Cβ. Mice are viable after knockout of either gene, suggesting that either isoform can perform the required physiological functions of this kinase [15,16]. To determine the functional consequences of depleting both of the Pka C subunits in RAW cells, we assessed the cAMP-induced phosphorylation of the vasodilator stimulated phosphoproteins (VASP), a known substrate of Pka [17]. In response to cAMP analogs, we observed a significant increase in phospho-VASP, which was unaffected in cells depleted of either Pka Cα or Pka Cβ (Fig. 4a). However, in cells with both Cα and Cβ knocked down, there was almost complete attenuation of VASP phosphorylation in response to cAMP (Fig. 4a). It is well established that Pka mediates many transcriptional effects, primarily through phosphorylation and activation of cAMP response element (CRE) binding protein (CREB) and cAMP response element modulator (CREM) [18]. Based on a recent microarray study in RAW cells [19], we identified several cAMP-dependent mRNAs and selected four targets for assessment in our Pka knockdown cell lines; Nr4a1, Nr4a2, Ctla2α and Ctla2β. Nr4a1/Nur77 and Nr4a2/Nurr1, members of the NR4A subfamily of orphan nuclear receptors, are potential transcriptional mediators in many cell types and have been shown to be regulated by cAMP [20]. Regulation of the transcription of Ctla2α and Ctla2β, cysteine protease inhibitors originally identified in cytotoxic T lymphocytes [21], has not been reported previously. None of these cAMP-dependent transcripts show any significant alteration of 8pCPT-cAMP induced increase in cells depleted for either Pka catalytic subunit, whereas depletion of both Pka Cα and Cβ leads to a marked decrease in transcript induction (Fig. 4b–e). These data provide the first evidence that Ctla2 transcription is PKA-dependent. More importantly, our data demonstrate the value of an RNAi platform which permits the depletion of multiple endogenous gene targets from a single viral infection of an intractable cell line. The system allows the demonstration of downstream biological consequences of the loss of a function redundantly performed by related proteins. Only upon effective depletion of both Pka C subunits is a phenotype observed (Fig. 4). There are numerous examples, especially in G-protein mediated signaling pathways, where mammalian cells express multiple proteins with redundant function, presumably to build robustness into the system. It is therefore noteworthy that the cells maintain a small degree of response in the recorded readouts in the dual knockdown cells (Fig. 4). Although this result could argue that an alternative cAMP effector, such as Epac, could contribute, it seems more likely that the residual Pka catalytic subunit (Fig. 3d+e) is capable of mediating the residual response. This observation supports the inherent robustness of this signaling pathway, and is consistent with the finding that the Pka Cα knockout mice remain viable despite certain cell types showing <10% of the wild type level of total PKA activity [16].

Using our multi-miR-shRNA expression platform, we demonstrate potent knockdown of up to three genes in transient expression studies (Fig. 1d–f) and three different pairs of endogenous genes after viral infection of a murine macrophage cell line (Fig. 3b–e). This approach has great potential for experimental strategies or clinical applications requiring depletion of multiple gene targets in cells with low transduction efficiency. It should be noted however, that the presence of increasing numbers of miR-shRNA cassettes in a viral transcript leads to a gradual reduction in viral titer (see Additional file 2), likely due to Drosha cleavage of the viral transcript during packaging. Despite this titer reduction, we were able to achieve viral titers of >1 × 10^6 ^pfu/ml from unconcentrated supernatants of lentivirus expressing three miR-shRNAs, well within the practical range for infection of most intractable cells.

**Figure 5:**
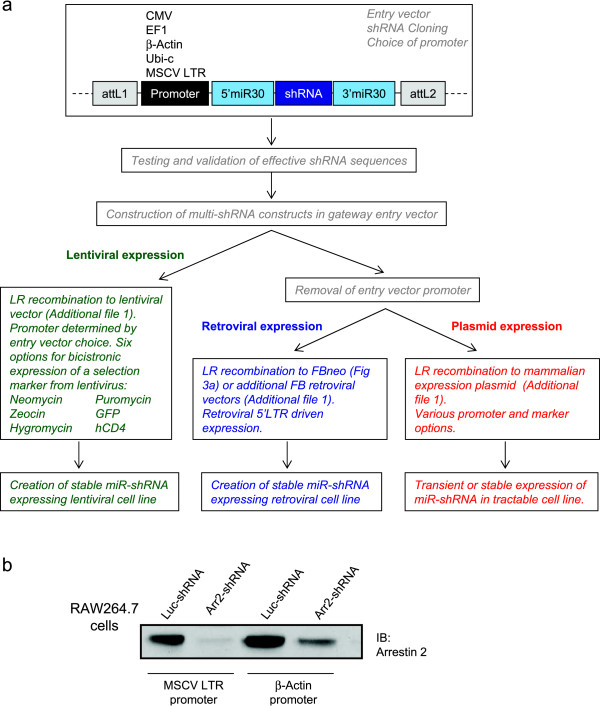
**miR-shRNA expression options**. **A) **Flow chart for expressing miR-shRNA in different expression platforms. Experimental details of the various steps are provided in methods. **B) **Comparison of MSCV LTR and beta actin promoters expressing arrestin 2 (Arr2) miR-shRNA or control luciferase (Luc) miR-shRNA in lentivirally infected RAW264.7 macrophages. Constructs used for lentivirus production are detailed in Additional file 3.

We engineered the vectors to be extremely versatile with a high degree of flexibility for downstream applications (Fig. 5a and Additional file 1). We created the initial shRNA cloning/testing entry vector with a choice of up to five interchangeable promoters (CMV, EF1, beta-actin, ubiquitin-c and MSCV 5'LTR), which can be combined with six different selection markers upon recombination to a selection of lentiviral vectors (Fig. 5a). This allows selection of the optimal promoter for a given cell system; for example, comparison of MSCV LTR and β-actin promoters driving the Arr2 miR-shRNA in RAW cells shows the former promotes more potent knockdown (Fig. 5b). Alternatively, the entry vector promoter can be removed after shRNA validation for recombination to a variety of expression platforms with existing promoters. These include 5'LTR-driven retroviral vectors or CMV/EF1-driven mammalian expression vectors which can then be used for creation of stable cell lines. These latter vectors also contain a broad selection of co-expressed fluorescent and/or drug selection markers (Additional file 1). Furthermore, the Gateway compatibility of these vectors allows the miR-shRNA cassettes to be recombined to any appropriately configured Gateway-ready expression system.

## Conclusion

We have developed an flexible cloning platform for generation of plasmid vectors and viruses expressing miR-shRNAs against multiple target genes. The ability to express multiple engineered miR-shRNA cassettes from a single transcript has been previously reported [5,11], and recent reports have shown that it can also be used to improve knockdown efficiency by expressing multiple miR-shRNA against the same target gene [22,23] and to promote multi-gene knockdown [22-24]. However, Refs.22 and 24 used intron-based expression of miR-shRNA cassettes from plasmids introduced into easily transfected cell lines, which is not compatible with the viral based approaches required with less tractable cell systems. To our knowledge, our data are the first to demonstrate functional consequences of depleting multiple endogenous target genes in mammalian cells using miR-shRNA where multi-gene knockdown is necessary to observe a phenotype (Fig. 4). We believe that the versatility of the vectors we describe here make them a valuable resource to the research community. Although we clone shRNAs into our entry vectors using BfuAI compatible linkers, we include Xho I and Eco RI cloning sites in the flanking miR30 sequence to allow subcloning of miR-shRNAs from popular whole genome libraries [2,7] into our plasmids (Fig. 2b). Any shRNA subcloned by either of these methods is then compatible for concatenation in a multi-miR-shRNA transcript (Fig. 1c).

## Availability and requirements

In accordance with the policy of the Alliance for Cellular Signaling (AfCS), all the vectors described in this study will be readily available through the American Type Culture Collection. Details will be provided at .

## Methods

### Reagents

8-Br-cAMP and 8-pCPT-cAMP were purchased from Calbiochem.

### Plasmid construction

Entry vectors for cloning miR-shRNA downstream of various promoters were created by excision of the U6 promoter from the pEN_hUmiRc2 plasmid [11] by SalI digest and ligation of SalI-flanked PCR products of the β-actin, CMV, EF1, MSCV LTR and Ubi-c promoters. Cloning of gene specific miR-shRNA into these entry vectors was by generation of BfuAI-compatible linkers as described previously [11]. For the lentiviral destination vectors, the Ubi-c promoter and GFP cDNA were removed from the pDSL_hpUG vector (ATCC# 10326371, AfCS# L06DDLHPUGXA) and replaced with cassettes including an IRES sequence followed by either GFP, truncated human CD4, neomycin, hygromycin, puromycin or zeocin. The pDS_FBneo retroviral destination vector was created by insertion of an attR site containing Gateway cassette (Invitrogen) into the multiple cloning site of the pFB-Neo plasmid (Stratagene). For the FBneo vectors containing fluorescent proteins, the attR cassette and fluorescent protein were subcloned as a single fragment into pFB-Neo from the mammalian expression plasmids described below. The FBhyg retroviral vectors were prepared in the same fashion after excision of the neomycin gene from pFB-Neo by ClaI/RsrII digest and insertion of the hygromycin resistance cDNA. The CMV promoter-containing destination vectors for mammalian co-expression of miR-shRNA with GFP derivatives were created by insertion of an attR site containing Gateway cassette (Invitrogen) into the multiple cloning site of the pEGFP-N1, pECFP-N1 and pEYFP-N1 vectors (Clontech). The pDS_X-mCherry vector was created in the same fashion from pcDNA-mCherry (derived from pRSET-B-mCherry, generously provided by Dr. Roger Tsien, University of California San Diego). The EF1 promoter versions of the GFP, CFP and YFP constructs were created in the same way after replacement of the CMV promoter by EF1 in the initial plasmids. The retroviral and lentiviral constructs containing various miR-shRNA (Additional file 3) were all created by LR recombination as described below.

### Cloning and validation of miR-shRNAs

Design and cloning of miR-shRNAs has been described previously [11]. Briefly, sequences were selected using the RNAi Codex algorithm [25], with 2–4 independent miR-shRNAs chosen for each target gene. Gene-specific sequences were cloned as BfuAI-compatible oligonucleotide linkers into the pEN_CmiRc2 vector (Additional files 1 and 3). The efficacy of the miR-shRNAs was validated by co-expression with YFP fusions of their cognate target cDNAs in HEK293 cells as previously described [26]. The YFP fusion constructs used for these experiments were obtained from the recently described AfCS plasmid collection [27]; pEX_EF1_YFP-Arrestin2; AfCS barcode A08XF063A1TK; atcc id 10374002, pEX_EF1_YFP-Grk2; AfCS barcode A08XK093A1TK; atcc id 9830392, pEX_EF1_YFP-Grk5; AfCS barcode A08XK213A1TK; atcc id 9891141, pEX_EF1_YFP-Gbeta2; AfCS barcode A08XF075A1TK; atcc id 9891160, pEX_EF1_YFP-PKACa; AfCS barcode A08XP020A1TK; atcc id 9891081 and pEX_EF1_YFP-PKACb; AfCS barcode A08XK074A1TK; atcc id 9830324. The most potent miR-shRNA identified by this method targeted the following murine sequences: arrestin 2; TCTCATAGAGCTTGACACCAAT, arrestin 3; TGCGGCTTATCATCAGAAAGGT, G-protein coupled receptor kinase 2; ACCGAGGAGAAGTGACCTTTGA, G-protein coupled receptor kinase 5; AGGCGGCAGCATCAAAGCAATT, G-protein β2; TGCTCATGTATTCCCACGACAA, Pka Cα; AGCCTATCCAGATCTATGAGAA and Pka Cβ; AAGGTTGTTAAGCTGAAGCAAA.

### Creation of constructs with multiple miR-shRNA cassettes

Constructs with multiple miR-shRNA cassettes were created by ligating SpeI/PstI fragments containing the desired downstream shRNA into an XbaI/PstI cut recipient plasmid containing the desired upstream shRNA(s) (Fig. 1c). Loss of the SpeI and XbaI sites permitted cloning of additional miR-shRNA via the same approach. The resulting constructs were validated by digestion with BsrGI, which has recognition sites in the attL1 and attL2 regions.

### Subcloning of miR-shRNA cassettes to different expression platforms

Constructs were designed to permit subcloning of miR-shRNA cassettes to different expression platforms by Gateway recombination. For lentiviral expression options, this involved direct LR recombination of the promoter-containing entry vector to any one of six lentiviral destination vectors with bicistronic expression of different markers or drug selection genes (Additional files 1 and 3). The destination vectors for either retroviral or mammalian plasmid expression have existing promoters, so for these platforms the promoter was removed from the entry vector prior to LR recombination. Entry vector promoters were removed either by BamHI/SpeI or DraI/SpeI digest (see Fig. 2a) and the vector backbone was religated after treatment with T4 DNA polymerase to flush the DNA termini. Promoter removal was confirmed in recombinant clones by BsrGI digest. Retroviral vectors for this study were created by LR recombination of miR-shRNA cassettes to pDS_FBneo (Additional files 1 and 3).

### Generation of viruses and creation of stable RAW264.7 cell lines

Production of lentiviruses and creation of stable lentiviral RAW cells was carried out as previously described [26]. Retrovirus was generated by transfection of the Phoenix 293T Amphotropic packaging cell line with miR-shRNA-expressing FBneo retroviral vectors. Briefly, Phoenix cells were cultured at 37°C, 5% CO2 in 100 mm tissue culture dishes (Corning) using DMEM (Gibco Invitrogen), 10% FBS (Gemini), 2 mM glutamine (Gibco Invitrogen), and 100 U/ml Penicillin with 100 μg/ml streptomycin (Gibco invitrogen). 10 μg per dish of retroviral vector diluted in Opti-MEM (Gibco Invitrogen) was transfected using Lipofectamine 2000 reagent (Invitrogen). Around 16 hours post-transfection, the medium on the Phoenix cells was replaced. Viral supernatant was harvested at 48 hours post-transfection, spun at 1200 rpm for 5 minutes and filtered through a 0.45 μm filter. Polybrene was added to a final concentration of 16 μg/ml, and equal volumes of viral supernantant were placed onto RAW264.7 cells plated in 6 well tissue culture plates (Corning) 2 hours prior to virus addition. Final polybrene concentration was 8 μg/ml. The medium on the RAW264.7 cells was replaced around 16 hours post-infection. 48 hours post-infection, RAW264.7 cells were re-seeded onto 100 mm dishes, and infected cells were selected using 500 μg/ml Geneticin (Gibco Invitrogen).

### Assessment of mRNA and protein expression levels

Knockdown of endogenous target mRNA and protein in stable cell lines was assessed from samples collected at least 14 days after viral infection, with at least one assessment carried out at >21 days. Detailed procedures for assessment of mRNA by quantitative real-time PCR (qRT-PCR) and protein by western blot have been described previously [26]. Sense and antisense amplification primers and probe primer sequences (where applicable) were: Arr2; 5'-GACTCCAGTAGACACCAATCTCAT-3'; 5'-ATCTGTTGTTGAGGTGCGGAG-3'; 5'-TexasRed-CGGTGCCGTCATCCTCTTCGTCCT-BHQ2-3', Arr3; 5'-GCCGACATTTGCCTCTTCAG-3'; 5'-CTAGGAGACACCTGGTCATCTTG-3'; 5'-TexasRed-CGCAGTACAAGTGTCCCGTGGCTC-BHQ2-3', Grk2; 5'-CCAGGAACTGTACCGCAACTT-3'; 5'-GGTCTGTTTCAGCATTGATGGTAT-3'; 5'- TexasRed-CTCTGCCACCACCTGTTGCCACCG-BHQ2-3', Grk5; 5'-ACGGTACCCTCTCACCAGAC-3'; 5'-TTTATTCGGTGGTTACAACTGGTC-3'; 5'-TexasRed-AGAAGTCAGCCTCCAGAACCGCCA-BHQ2-3', PkaCα; 5'-CTCCCACCCTCCAAACTGTC-3'; 5'-GACAGGGTCAGTTGGCTACC-3'; 5'-FAM-ACCCTCCCCAAACACCCTCCTCAC-BHQ1-3', Pka Cβ; 5'- GATGGAATGTCTTGTCAGCATGG-3'; 5'-TCGTCCAGGAGTCCTCACTG-3'; 5'-TexasRed-AGGCGCTCTGACTCACTGCTGCAT-BHQ2-3', Nr4a1; 5'-ACAGCTTGGGTGTTGATGTTCC-3'; 5'-GCCATGTGCTCCTTCAGACAG-3', Nr4a2; 5'-ACAGTTTAAAAGGCCGGAGAGG-3'; 5'-GTCATTGCCGGATTGGAATCG-3', Ctla2α; 5'-TGTGGCTTGACTGGTAAC-3'; 5'-CAGCATCATTCCTCTCATAC-3', Ctla2β; 5'-GCATTGTCTTGGGAGTCTTC-3'; 5'-ACAGTGTTGATCTTATATGAGTTC-3' and the β-actin reference; 5'-TCCATGAAATAAGTGGTTACAGGA-3'; 5'-CAGAAGCAATGCTGTCACCTT-3'; 5'-HEX-TCCCTCACCCTCCCAAAAGCCACC-BHQ1-3'. The following antisera were used; anti-YFP (BD Clontech #8371-2), anti-Pka Cα (Cell Signaling Technology, #4782), anti-Pka Cβ (Santa Cruz Biotechnology, sc-904) and Arr2 (A1CT #140; generously provided by Dr. Robert Lefkowitz, Duke University).

### Phosphoprotein detection by western blot

A detailed protocol for detection of phosphoproteins in extracts from stimulated RAW cells can be found on the AfCS website [28] (PP00000177 and PP00000181). Following this protocol, RAW cells were stimulated for 10 min with 100 μM 8Br-cAMP or 8pCPT-cAMP, and phospho-VASP levels were assessed using an antibody from Cell Signaling Technology (#3111).

## Authors' contributions

XZ generated and assayed the PKA knockdown cell lines and co-wrote the manuscript, LAS established the efficacy of concatenated miR-shRNAs and generated the Arrestin and Grk knockdown lines, MSC determined the transcriptional consequences of PKA knockdown, JL, JRZ, EAW and CK designed and constructed vectors and developed effective miR-shRNAs, MIS participated in the design of the study, IDCF conceived of the study, participated in its design and coordination and co-wrote the manuscript. All authors read and approved the final manuscript.

## Supplementary Material

Additional file 1 Details of parent plasmids described in this study available from the ATCC. A table including details of plasmids for cloning and expression of miR-shRNA from a variety of expression platforms. Click here for file

Additional file 2 Effect of multiple miR-shRNA cassettes on lentiviral titer. The data show the relative titer of lentiviruses containing increasing numbers of miR-shRNA. Click here for file

Additional file 3 Details of miR-shRNA-containing plasmids described in this study available from the ATCC. A table including details of plasmids containing validated miR-shRNAs against select target genes used in this study. Click here for file

## References

[B1] Montgomery MK (2006). RNA interference: unraveling a mystery. Nat Struct Mol Biol.

[B2] Chang K, Elledge SJ, Hannon GJ (2006). Lessons from Nature: microRNA-based shRNA libraries. Nat Methods.

[B3] Amarzguioui M, Rossi JJ, Kim D (2005). Approaches for chemically synthesized siRNA and vector-mediated RNAi. FEBS Lett.

[B4] Zeng Y, Wagner EJ, Cullen BR (2002). Both natural and designed micro RNAs can inhibit the expression of cognate mRNAs when expressed in human cells. Mol Cell.

[B5] Zeng Y, Cullen BR (2003). Sequence requirements for micro RNA processing and function in human cells. Rna.

[B6] Boden D, Pusch O, Silbermann R, Lee F, Tucker L, Ramratnam B (2004). Enhanced gene silencing of HIV-1 specific siRNA using microRNA designed hairpins. Nucleic Acids Res.

[B7] Silva JM, Li MZ, Chang K, Ge W, Golding MC, Rickles RJ, Siolas D, Hu G, Paddison PJ, Schlabach MR, Sheth N, Bradshaw J, Burchard J, Kulkarni A, Cavet G, Sachidanandam R, McCombie WR, Cleary MA, Elledge SJ, Hannon GJ (2005). Second-generation shRNA libraries covering the mouse and human genomes. Nat Genet.

[B8] Stegmeier F, Hu G, Rickles RJ, Hannon GJ, Elledge SJ (2005). A lentiviral microRNA-based system for single-copy polymerase II-regulated RNA interference in mammalian cells. Proc Natl Acad Sci U S A.

[B9] Dickins RA, Hemann MT, Zilfou JT, Simpson DR, Ibarra I, Hannon GJ, Lowe SW (2005). Probing tumor phenotypes using stable and regulated synthetic microRNA precursors. Nat Genet.

[B10] Zhou H, Xia XG, Xu Z (2005). An RNA polymerase II construct synthesizes short-hairpin RNA with a quantitative indicator and mediates highly efficient RNAi. Nucleic Acids Res.

[B11] Shin KJ, Wall EA, Zavzavadjian JR, Santat LA, Liu J, Hwang JI, Rebres R, Roach T, Seaman W, Simon MI, Fraser ID (2006). A single lentiviral vector platform for microRNA-based conditional RNA interference and coordinated transgene expression. Proc Natl Acad Sci U S A.

[B12] Lagos-Quintana M, Rauhut R, Lendeckel W, Tuschl T (2001). Identification of novel genes coding for small expressed RNAs. Science.

[B13] Lau NC, Lim LP, Weinstein EG, Bartel DP (2001). An abundant class of tiny RNAs with probable regulatory roles in Caenorhabditis elegans. Science.

[B14] Gilman AG, Simon MI, Bourne HR, Harris BA, Long R, Ross EM, Stull JT, Taussig R, Bourne HR, Arkin AP, Cobb MH, Cyster JG, Devreotes PN, Ferrell JE, Fruman D, Gold M, Weiss A, Stull JT, Berridge MJ, Cantley LC, Catterall WA, Coughlin SR, Olson EN, Smith TF, Brugge JS, Botstein D, Dixon JE, Hunter T, Lefkowitz RJ, Pawson AJ, Sternberg PW, Varmus H, Subramaniam S, Sinkovits RS, Li J, Mock D, Ning Y, Saunders B, Sternweis PC, Hilgemann D, Scheuermann RH, DeCamp D, Hsueh R, Lin KM, Ni Y, Seaman WE, Simpson PC, O'Connell TD, Roach T, Simon MI, Choi S, Eversole-Cire P, Fraser I, Mumby MC, Zhao Y, Brekken D, Shu H, Meyer T, Chandy G, Heo WD, Liou J, O'Rourke N, Verghese M, Mumby SM, Han H, Brown HA, Forrester JS, Ivanova P, Milne SB, Casey PJ, Harden TK, Arkin AP, Doyle J, Gray ML, Meyer T, Michnick S, Schmidt MA, Toner M, Tsien RY, Natarajan M, Ranganathan R, Sambrano GR (2002). Overview of the Alliance for Cellular Signaling. Nature.

[B15] Howe DG, Wiley JC, McKnight GS (2002). Molecular and behavioral effects of a null mutation in all PKA C beta isoforms. Mol Cell Neurosci.

[B16] Skalhegg BS, Huang Y, Su T, Idzerda RL, McKnight GS, Burton KA (2002). Mutation of the Calpha subunit of PKA leads to growth retardation and sperm dysfunction. Mol Endocrinol.

[B17] Butt E, Abel K, Krieger M, Palm D, Hoppe V, Hoppe J, Walter U (1994). cAMP- and cGMP-dependent protein kinase phosphorylation sites of the focal adhesion vasodilator-stimulated phosphoprotein (VASP) in vitro and in intact human platelets. J Biol Chem.

[B18] Zambon AC, Zhang L, Minovitsky S, Kanter JR, Prabhakar S, Salomonis N, Vranizan K, Dubchak I, Conklin BR, Insel PA (2005). Gene expression patterns define key transcriptional events in cell-cycle regulation by cAMP and protein kinase A. Proc Natl Acad Sci U S A.

[B19] Zhu X, Chang MS, Hsueh RC, Taussig R, Smith KD, Simon MI, Choi S (2006). Dual ligand stimulation of RAW 264.7 cells uncovers feedback mechanisms that regulate TLR-mediated gene expression. J Immunol.

[B20] Pei L, Waki H, Vaitheesvaran B, Wilpitz DC, Kurland IJ, Tontonoz P (2006). NR4A orphan nuclear receptors are transcriptional regulators of hepatic glucose metabolism. Nat Med.

[B21] Denizot F, Brunet JF, Roustan P, Harper K, Suzan M, Luciani MF, Mattei MG, Golstein P (1989). Novel structures CTLA-2 alpha and CTLA-2 beta expressed in mouse activated T cells and mast cells and homologous to cysteine proteinase proregions. Eur J Immunol.

[B22] Chung KH, Hart CC, Al-Bassam S, Avery A, Taylor J, Patel PD, Vojtek AB, Turner DL (2006). Polycistronic RNA polymerase II expression vectors for RNA interference based on BIC/miR-155. Nucleic Acids Res.

[B23] Sun D, Melegari M, Sridhar S, Rogler CE, Zhu L (2006). Multi-miRNA hairpin method that improves gene knockdown efficiency and provides linked multi-gene knockdown. Biotechniques.

[B24] Xia XG, Zhou H, Xu Z (2006). Multiple shRNAs expressed by an inducible pol II promoter can knock down the expression of multiple target genes. Biotechniques.

[B25] The RNAi Central website. http://katahdin.cshl.org:9331/portal/scripts/main2.pl.

[B26] Fraser I, Liu W, Rebres R, Roach T, Zavzavadjian J, Santat L, Liu J, Wall E, Mumby M (2007). The use of RNA interference to analyze protein phosphatase function in Mammalian cells. Methods Mol Biol.

[B27] Zavzavadjian JR, Couture S, Park WS, Whalen J, Lyon S, Lee G, Fung E, Mi Q, Liu J, Wall E, Santat L, Dhandapani K, Kivork C, Driver A, Zhu X, Chang MS, Randhawa B, Gehrig E, Bryan H, Verghese M, Maer A, Saunders B, Ning Y, Subramaniam S, Meyer T, Simon MI, O'Rourke N, Chandy G, Fraser ID (2007). The alliance for cellular signaling plasmid collection: a flexible resource for protein localization studies and signaling pathway analysis. Mol Cell Proteomics.

[B28] The AfCS website protocols. http://www.signaling-gateway.org/data/ProtocolLinks.html.

[B29] The AfCS sponsors. http://www.signaling-gateway.org/aboutus/sponsors.html.

