# Supplementary material for: A versatile approach to multiple gene RNA interference using microRNA-based short hairpin RNAs
Source: BMC Mol Biol. 2007 Oct 30;8:98. doi: 10.1186/1471-2199-8-98 (PMC2194719; doi:10.1186/1471-2199-8-98)
Supplement: Additional file 1 — Details of parent plasmids described in this study available from the ATCC. A table including details of plasmids for cloning and expression of miR-shRNA from a variety of expression platforms. [file 1471-2199-8-98-S1.pdf]

Additional file 1

Details of parent plasmids described in this study available from the ATCC. Entry vectors for cloning of miR-shRNA driven by different promoters and destination vectors for expression of miR-shRNA from lentivirus, retrovirus or mammalian plasmids are shown. Both AfCS barcodes and ATCC IDs are shown for locating plasmid information online. All the plasmids below are included in plate MBA-330.

AfCS plasmid db: http://www.signaling-gateway.org/data/plasmid/;

ATCC clone search: http://www.atcc.org/common/catalog/molecular/index.cfm.

Construct name	***Vector details***	***AfCS barcode***	***ATCC id***
pEN_CmiRc2	Entry vector; shRNA cloning/validation; CMV promoter	C12ECMIRC2AG	MBA-284
pEN_cA4miRc	Entry vector; shRNA cloning/validation; Chicken beta actin promoter	P20ECA4MIRAG	MBA-285
pEN_EmiRc3	Entry vector; shRNA cloning/validation; EF1-alpha promoter	P34EEFMIR3AG	MBA-286
pEN_LmiRc3	Entry vector; shRNA cloning/validation; MSCV LTR promoter	P35ELTMIR3AG	MBA-287
pEN_UBmiRc3	Entry vector; shRNA cloning/validation; Ubi-c promoter	P36EUBMIR3AG	MBA-288
pDSL_hpIC	Lentiviral Expression; Promoterless; Gateway cassette u/s of IRES-CD4	L67DDLHPICXA	MBA-289
pDSL_hpIG	Lentiviral Expression; Promoterless; Gateway cassette u/s of IRES-GFP	L68DDLHPIGXA	MBA-290
pDSL_hpIH	Lentiviral Expression; Promoterless; Gateway cassette u/s of IRES-Hygromycin	L73DDLHPIHXA	MBA-291
pDSL_hpIN	Lentiviral Expression; Promoterless; Gateway cassette u/s of IRES-Neo	L66DDLHPINXA	MBA-292
pDSL_hpIP	Lentiviral Expression; Promoterless; Gateway cassette u/s of IRES-Puromycin	L75DDLHPIPXA	MBA-293
pDSL_hpIZ	Lentiviral Expression; Promoterless; Gateway cassette u/s of IRES-Zeocin	L74DDLHPIZXA	MBA-294
pDS_FBneo	Retroviral Expression; MMLV 5'LTR promoter; Bicistronic neomycin expression	C31DDSFBNEXA	MBA-295
pDS-FBneo_X-CFP	Retroviral Expression; MMLV 5'LTR promoter; C-terminal CFP; Bicistronic neomycin expression	C22DFBNXCFNA	MBA-296
pDS-FBneo_X-mCH	Retroviral Expression; MMLV 5'LTR promoter; C-terminal mCherry; Bicistronic neomycin expression	C30DFBNXCHNA	MBA-297
pDS-FBneo_X-YFP	Retroviral Expression; MMLV 5'LTR promoter; C-terminal YFP; Bicistronic neomycin expression	C23DFBNXYFNA	MBA-298
pDS_FBhyg	Retroviral Expression; MMLV 5'LTR promoter; Bicistronic hygromycin expression	C33DDSFBHYXA	MBA-299
pDS-FBhyg_X-CFP	Retrovira Expression; MMLV 5'LTR promoter; C-terminal CFP; Bicistronic hygromycin expression	C18DFBHXCFNA	MBA-300
pDS-FBhyg_X-mCH	Retroviral Expression; MMLV 5'LTR promoter; C-terminal mCherry; Bicistronic hygromycin expression	C28DFBHXCHNA	MBA-301
pDS-FBhyg_X-YFP	Retroviral Expression; MMLV 5'LTR promoter; C-terminal YFP; Bicistronic hygromycin expression	C19DFBHXYFNA	MBA-302
pDS_XB-GFP	Mammalian Expression; CMV promoter; C-terminal GFP	A25DCXBGFPNK	10326345
pDS_XB-CFP	Mammalian Expression; CMV promoter; C-terminal CFP	A02DCXBCFPNK	10326346
pDS_XB-YFP	Mammalian Expression; CMV promoter; C-terminal YFP	A01DCXBYFPNK	10326347
pDS_X-mCherry	Mammalian Expression; CMV promoter; C-terminal mCherry	C25DCXMCHYNA	MBA-303
pDS_EF1-XB-GFP	Mammalian Expression; EF1 promoter; C-terminal GFP	A27DFXBGFPNK	10326349
pDS_EF1-XB-CFP	Mammalian Expression; EF1 promoter; C-terminal CFP	A06DFXBCFPNK	10326353
pDS_EF1-XB-YFP	Mammalian Expression; EF1 promoter; C-terminal YFP	A05DFXBYFPNK	10326350
