# Supplementary material for: A versatile approach to multiple gene RNA interference using microRNA-based short hairpin RNAs
Source: BMC Mol Biol. 2007 Oct 30;8:98. doi: 10.1186/1471-2199-8-98 (PMC2194719; doi:10.1186/1471-2199-8-98)

Additional file 2

Effect of multiple miR-shRNA cassettes on lentiviral titer. **a:** Schematic of the lentiviral vector used to assess effect of multiple miR-shRNA cassettes on lentiviral titer. **b:** Relative viral titer of lentiviruses co-expressing up to three miR-shRNA cassettes. Average titer of unconcentrated control virus was 1 x 10^7 pfu/ml.

a

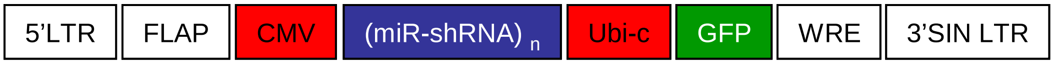

b

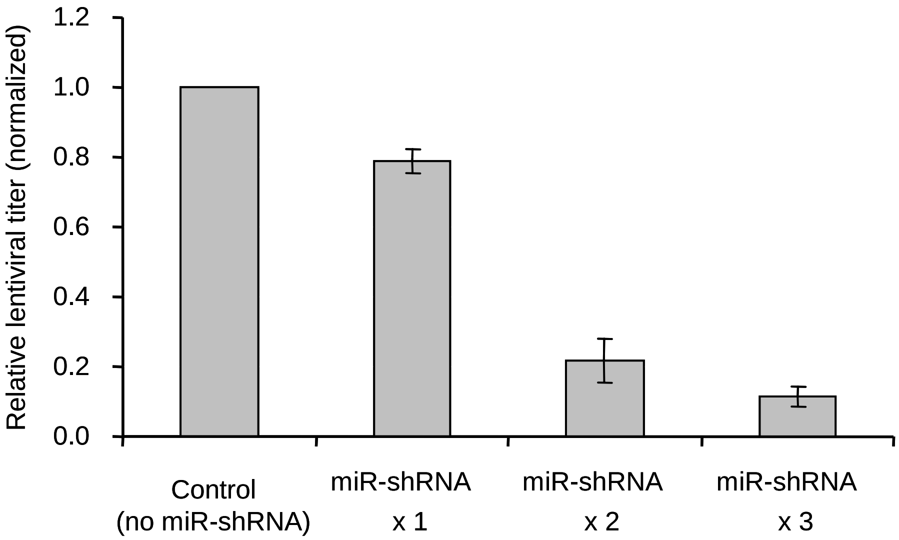

Supplement: Additional file 2 — Effect of multiple miR-shRNA cassettes on lentiviral titer. The data show the relative titer of lentiviruses containing increasing numbers of miR-shRNA. [file 1471-2199-8-98-S2.pdf]
