# Supplementary material for: A versatile approach to multiple gene RNA interference using microRNA-based short hairpin RNAs
Source: BMC Mol Biol. 2007 Oct 30;8:98. doi: 10.1186/1471-2199-8-98 (PMC2194719; doi:10.1186/1471-2199-8-98)
Supplement: Additional file 3 — Details of miR-shRNA-containing plasmids described in this study available from the ATCC. A table including details of plasmids containing validated miR-shRNAs against select target genes used in this study. [file 1471-2199-8-98-S3.pdf]

Additional file 3

Details of miR-shRNA-containing plasmids described in this study available from the ATCC. Entry, retroviral and lentiviral vectors with either single or multiple miR-shRNA against select genes are shown. Both AfCS barcodes and ATCC IDs are shown for locating plasmid information online. All the plasmids below are included in plate MBA-330.

AfCS plasmid db: http://www.signaling-gateway.org/data/plasmid/;

ATCC clone search: http://www.atcc.org/common/catalog/molecular/index.cfm.

Construct name	***Vector details***	***AfCS barcode***	***ATCC id***
pEN_CmiR-Luc-A	Entry vector; CMV-driven miR-shRNA	C12NX023HAAG	MBA-304
pEN_CmiR-Arr2-5D	Entry vector; CMV-driven miR-shRNA	C12NF0635DAG	MBA-305
pEN_hU6miR-Arr3-E	Entry vector; U6-driven miR-shRNA	P13NS055HEAG	MBA-306
pEN_CmiR-Grk2-H	Entry vector; CMV-driven miR-shRNA	C12NK093HHAG	MBA-307
pEN_hU6miR-Grk5-F	Entry vector; U6-driven miR-shRNA	P13NK213HFAG	MBA-308
pEN_CmiR-Gb2-K	Entry vector; CMV-driven miR-shRNA	C12NF075HKAG	MBA-309
pEN_CmiR-Pkaca-A	Entry vector; CMV-driven miR-shRNA	C12NK083HAAG	MBA-310
pEN_CmiR-Pkacb-A	Entry vector; CMV-driven miR-shRNA	C12NK074HAAG	MBA-311
pEN_CmiR-Arr2-5D_Gb2-K	Entry vector; CMV-driven double miR-shRNA	M0330CMV2SHG	MBA-312
pEN_CmiR-Grk2-H_Arr2-5D	Entry vector; CMV-driven double miR-shRNA	M0331CMV2SHG	MBA-313
pEN_CmiR-Grk2-H_Gb2-K	Entry vector; CMV-driven double miR-shRNA	M0332CMV2SHG	MBA-314
pEN_CmiR-Gb2-K_Arr2-5D_Grk2-H	Entry vector; CMV-driven triple miR-shRNA	M0333CMV3SHG	MBA-315
pFB-Neo-Luc-A	Retrovirus; MoMuLV 5'LTR-driven miR-shRNA-IRES-Neo	M0172PFB1SHA	MBA-316
pFB-Neo-Arr2-5D	Retrovirus; MoMuLV 5'LTR-driven miR-shRNA-IRES-Neo	M0169PFB1SHA	MBA-317
pFB-Neo-Arr3-E	Retrovirus; MoMuLV 5'LTR-driven miR-shRNA-IRES-Neo	M0171PFB1SHA	MBA-318
pFB-Neo-Arr2-5D_Arr3-E	Retrovirus; MoMuLV 5'LTR-driven double miR-shRNA-IRES-Neo	M0170PFB2SHA	MBA-319
pFB-Neo-Grk2-H	Retrovirus; MoMuLV 5'LTR-driven miR-shRNA-IRES-Neo	M0204PFB1SHA	MBA-320
pFB-Neo-Grk5-F	Retrovirus; MoMuLV 5'LTR-driven miR-shRNA-IRES-Neo	M0205PFB1SHA	MBA-321
pFB-Neo-Grk2-H_Grk5-F	Retrovirus; MoMuLV 5'LTR-driven double miR-shRNA-IRES-Neo	M0208PFB2SHA	MBA-322
pFB-Neo-Pkaca-A	Retrovirus; MoMuLV 5'LTR-driven miR-shRNA-IRES-Neo	M0327PFB1SHA	MBA-323
pFB-Neo-Pkacb-A	Retrovirus; MoMuLV 5'LTR-driven miR-shRNA-IRES-Neo	M0328PFB1SHA	MBA-324
pFB-Neo-Pkaca-A_Pkacb-A	Retrovirus; MoMuLV 5'LTR-driven double miR-shRNA-IRES-Neo	M0329PFB2SHA	MBA-325
pLX_LmiR_Luc-A_IN	Lentivirus; MoMuLV 5'LTR-driven miR-shRNA-IRES-Neo	M0251LLA1SHA	MBA-326
pLX_LmiR_Arr2-5D_IN	Lentivirus; MoMuLV 5'LTR-driven miR-shRNA-IRES-Neo	M0252PLA1SHA	MBA-327
pLX_cA4miR_Luc-A_IN	Lentivirus; beta actin promoter-driven miR-shRNA-IRES-Neo	M0254PLL1SHA	MBA-328
pLX_cA4miR_Arr2-5D_IN	Lentivirus; beta actin promoter-driven miR-shRNA-IRES-Neo	M0253PLL1SHA	MBA-329
